# Draft Genome Sequences of *Tetragenococcus muriaticus* Strains 3MR10-3 and PMC-11-5 Isolated from Thai Fish Sauce during Natural Fermentation

**DOI:** 10.1128/genomeA.00198-17

**Published:** 2017-04-13

**Authors:** Chokchai Chuea-nongthon, Sureelak Rodtong, Jirawat Yongsawatdigul, James L. Steele

**Affiliations:** a School of Microbiology, Institute of Science, Suranaree University of Technology, Nakhon Ratchasima, Thailand; b School of Food Technology, Institute of Agricultural Technology, Suranaree University of Technology, Nakhon Ratchasima, Thailand; c Microbial Cultures Research Center for Food and Bioplastics Production, Suranaree University of Technology, Nakhon Ratchasima, Thailand; d Department of Food Science, University of Wisconsin-Madison, Madison, Wisconsin, USA

## Abstract

*Tetragenococcus muriaticus* strains 3MR10-3 and PMC-11-5 are homofermentative halophilic lactic acid bacteria isolated from Thai fish sauce during natural fermentation. Their draft genomes were sequenced. Our interest in these organisms is related to their impact on fish sauce flavor and their high osmotolerance.

## GENOME ANNOUNCEMENT

Halophilic lactic acid bacteria in the genus *Tetragenococcus*, including *T. muriaticus* and *T. halophilus*, have been reported to be isolated from fish sauce (1–4) and to play important roles in flavor development of the sauce (3, 4). Fish sauce is a fermented clear brownish liquid condiment with salty taste and distinct aroma and flavor (5, 6). Herein, we present the genomic sequences of two *T. muriaticus* strains, 3MR10-3 and PMC-11-5, which were isolated from fish sauce processed in eastern Thailand.

Genomic DNA was prepared from cultures propagated in De Man, Rogosa, and Sharpe (MRS) broth containing 0.5% CaCO_3_ and 5% NaCl, which had been incubated at 30°C for 48 h. The genomic DNA was prepared using the cetyltrimethylammonium bromide (CTAB) method (DOE Joint Genome Institute, CA) and subjected to genomic sequencing using 454 GS FLX Titanium paired-end sequencing platform (Life Technologies, Thermo Fisher Scientific, Inc., Waltham, MA, USA) at the Biotechnology Center, University of Wisconsin-Madison. The nucleotide sequences were assembled using the GS *de novo* assembler version 2.6 (Life Technologies, Thermo Fisher Scientific, Inc.). Functional annotation was performed by the Rapid Annotations using Subsystems Technology (RAST) server (7).

The genome sizes of *T. muriaticus* strains 3MR10-3 and PMC-11-5 were 2,080,407 and 2,103,938 bp with a total of 322 and 376 contigs, respectively. The G+C contents of the two strains, 3MR10-3 and PMC-11-5, were 35.96 and 36.03%, respectively, and the total numbers of predicted coding sequences (CDSs) were 2,252 and 2,626 CDSs, with 52 and 50 tRNAs and five and three rRNAs, respectively.

The genomes of strains 3MR10-3 and PMC-11-5 were examined for genes potentially involved in osmotolerance or fish sauce flavor development. For osmotolerance, we focused on genes coding for transporters responsible for inorganic ion or compatible solute uptake from hypersaline environments (8, 9). This analysis resulted in the identification of genes responsible for K^+^ transport, an ABC-type proline/glycine betaine transporter, a glycine betaine/choline transporter, and a choline/carnitine/betaine transporter. To identify genes likely involved in fish sauce flavor development, we focused upon genes related to nitrogen metabolism, as nitrogen metabolism has been linked to flavor development in a number of different fermented foods (10). Genes encoding transporters associated with free amino acid, di-/tripeptide, and oligopeptide uptake were identified and likely are responsible for the transport of extracellular protein hydrolysis products (11). In addition, genes encoding a variety of intracellular peptidases were identified, and these enzymes would be capable of converting the transported peptides into free amino acids. Enzymes involved in converting the intracellular amino acids into flavor compounds, such as several aminotransferases, were also identified. Finally, two key enzyme-encoding genes involved in volatile compound production, including alcohol dehydrogenase (*adh*), butanol dehydrogenase (*bdh*), and branched-chain alpha-keto acid dehydrogenase (*kadh*), were also identified. However, genes encoding cystathionine lyase (*met*C) and histidine decarboxylase (*hdc*), which generate sulfur-containing compounds and histamine, respectively, were not detected. These results will allow for further investigations into the mechanisms of osmotolerance and flavor formation by these organisms.

### Accession number(s).

The whole-genome shotgun (WGS) projects have been deposited at DDBJ/EMBL/GenBank under two accession numbers comprising the *Tetragenococcus muriaticus* 3MR10-3 WGS project having the project accession JPVT00000000, in which the first version of the project has the accession number JPVT01000000 and consists of sequences JPVT01000001 to JPVT01000302; and the *Tetragenococcus muriaticus* PMC-11-5 WGS project, having the project accession JPVU00000000, in which the first version of the project has the accession number JPVU01000000, and consists of sequences JPVU01000001 to JPVU01000354.
